# Identification of an indole biodegradation gene cluster from *Providencia rettgeri* and its contribution in selectively biosynthesizing Tyrian purple

**DOI:** 10.3389/fbioe.2022.1109929

**Published:** 2023-01-10

**Authors:** Feifei Li, Huaxiang Deng, Biming Zhong, Banlai Ruan, Xixi Zhao, Xiaozhou Luo

**Affiliations:** ^1^ School of Life Sciences, Inner Mongolia University, Hohhot, China; ^2^ Shenzhen Key Laboratory for the Intelligent Microbial Manufacturing of Medicines, Shenzhen Institute of Advanced Technology, Chinese Academy of Sciences, Shenzhen, China; ^3^ CAS Key Laboratory of Quantitative Engineering Biology, Shenzhen Institute of Synthetic Biology, Shenzhen Institute of Advanced Technology, Chinese Academy of Sciences, Shenzhen, China; ^4^ Center for Synthetic Biochemistry, Shenzhen Institute of Synthetic Biology, Shenzhen Institute of Advanced Technology, Chinese Academy of Sciences, Shenzhen, China

**Keywords:** Tyrian purple, *Providencia rettgeri*, indole biodegradation gene cluster, selective Tyrian purple producing, monooxygenase

## Abstract

Tyrian purple, mainly composed of 6, 6′-dibromoindigo, is a precious dye extracted from sea snails. In this study, we found Tyrian purple can be selectively produced by a bacterial strain GS-2 when fed with 6-bromotryptophan in the presence of tryptophan. This GS-2 strain was then identified as *Providencia rettgeri* based on bacterial genome sequencing analysis. An indole degradation gene cluster for indole metabolism was identified from this GS-2 strain. The heterologous expression of the indole degradation gene cluster in *Escherichia coli* BL21 (DE3) and *in vitro* enzymatic reaction demonstrated that the indole biodegradation gene cluster may contribute to selectively biosynthesizing Tyrian purple. To further explore the underlying mechanism of the selectivity, we explored the intermediates in this indole biodegradation pathway using liquid chromatography electrospray ionization quadrupole time-of-flight mass spectrometry (LC-ESI-QTOF-MS/MS), which indicated that the indole biodegradation pathway in *Providencia rettgeri* is the catechol pathway. Interestingly, the monooxygenase *GS*-C co-expressed with its corresponding reductase *GS*-D in the cluster has better activity for the biosynthesis of Tyrian purple compared with the previously reported monooxygenase from *Methylophaga aminisulfidivorans* (MaFMO) or *Streptomyces cattleya* cytochrome P450 enzyme (CYP102G4). This is the first study to show the existence of an indole biodegradation pathway in *Providencia rettgeri*, and the indole biodegradation gene cluster can contribute to the selective production of Tyrian purple.

## 1 Introduction

Tyrian purple, known as royal purple, and mainly composed of 6, 6′-dibromoindigo, is an ancient dye extracted from the murex shellfish (Ngangbam et al., 2015; Lee et al., 2021). Tyrian purple has a range of striking purple to red, color-fast and resistance to fading, and also has a promising application in dye-sensitized solar cells, functional polymers, and conductive materials (Głowacki et al., 2012; Głowacki et al., 2013; Guo et al., 2015; Kim et al., 2018; Schnepel et al., 2021). It is very difficult to obtain the dye in large quantities from natural sources as it requires euthanizing 12,000 snails per 1.4 g of dye (Mcgovern and Michel, 1990). In addition, due to the difficulty in chemical or biological synthesis, there is still no method for its industrial production (Wolk and Frimer, 2010). Biocatalysis has emerged as an alternative for sustainable synthesis of Tyrian purple from a natural substrate through microbial fermentation, but the selectivity issue in enzymatic tryptophan and bromotryptophan degradation becomes an obstacle for large-scale biosynthesis. Recently, Lee et al. presented an alternative 6, 6′-dibromoindigo production strategy in *E. coli* using tryptophan 6-halogenase from *Streptomyces toxytricini* (SttH), tryptophanase from *E. coli* (TnaA), and monooxygenase MaFMO.

**FIGURE 1:**
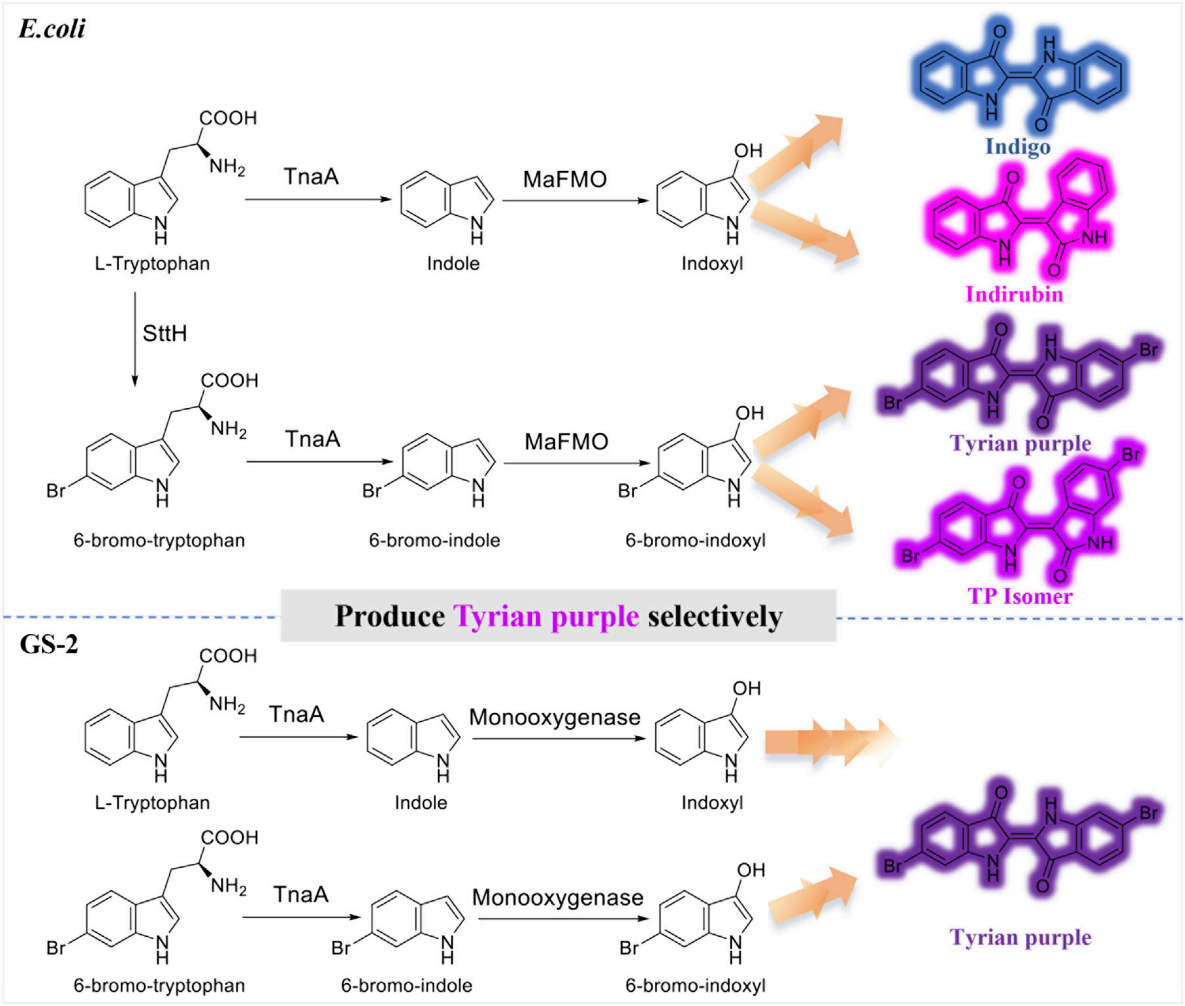
Production of indigo, indirubin, Tyrian purple, and TP isomer from tryptophan or 6-bromo-tryptophan: ***E. coli***, expressing tryptophan 6-halogenase SttH, and flavin-containing monooxygenase MaFMO from *Methylophaga aminisulfidivorans* in *E. coli*. **GS-2**, in the bacterial strain GS-2.

It is often not feasible to obtain pure Tyrian purple by biosynthesis. As TnaA is effective enough to convert tryptophan or its halogenated derivative into the corresponding indoles, and MaFMO catalyzes the hydroxylation of indoles (indole or 6-bromoindole) to 2-hydroxyindoles and 3-hydroxyindoles (Kim et al., 2019), the biosynthesis of Tyrian purple using three enzymes in *E. coli* would produce indigo and indirubin (isomer of indigo), Tyrian purple, and 6, 6′-dibromoindirubin (TP isomer, Tyrian purple isomer), simultaneously (Figure 1), that is, TnaA competes with SttH for tryptophan, both TnaA and MaFMO are lack of selectivity that lead to the production of many byproducts, which are the limitations of Tyrian purple biosynthesis. To overcome this TnaA competes with SttH for tryptophan issue, Lee et al. introduced a consecutive two-cell reaction system to overproduce regiospecifically brominated precursors of 6, 6′-dibromoindigo by spatiotemporal separation of bromination and bromotryptophan degradation (Lee et al., 2021). These approaches led to 315.0 mg L^−1^ 6, 6′-dibromoindigo production from 2.5 mM tryptophan. However, the final product is impure and the two-cell reaction process is still too inefficient and uneconomical to be feasible on an industrial scale for the biological synthesis of Tyrian purple.

Nevertheless, indoles are a typical class of N-heterocyclic aromatic pollutants and are widespread in our daily products and natural environment. In microbial communities, more than 85 bacterial species, including *Escherichia coli, Vibrio cholera*, and *Providencia rettgeri*, can catalyze tryptophan to indole by tryptophanase (TnaA) (O’hara et al., 2000; Lee and Lee, 2010; Ngangbam et al., 2015). However, indole and its derivatives at high concentrations are mutagens and carcinogens that exhibit toxic activity on microorganisms and animals (Lin et al., 2015; Tomberlin et al., 2017; Li et al., 2021). To defend against the toxicity of indole, many bacteria have established enzymatic detoxification systems, which is the oxidation of indole to insoluble non-toxic indigoid pigments or use the biodegradation mechanisms (Doukyu and Aono, 1997; Bhushan et al., 2000; Kim et al., 2003; Fukuoka et al., 2015; Zhang et al., 2018). A number of indole-degrading bacterial microorganisms and bacterial consortia were reported previously. *Acinetobacter*, *Alcaligenes*, *Burkholderia*, *Pseudomonas*, and *Cupriavidus* are the most extensively investigated indole-degrading bacterial genera (Yin et al., 2005; Kim et al., 2016). There are several reports on the identification of the indole biodegradation gene cluster and using it for the biological production of indigo in *E. coli*, but there are few reports on its application in the biosynthesis of indigoid dyes. Lin et al. identified an indigo-producing oxygenase *iacA* in *Acinetobacter baumannii* and that the iac gene cluster of *A. baumannii* is involved in indole 3-acetic acid degradation (Lin et al., 2012). Qu et al. isolated and unveiled the biotransformation mechanism of indole in *Cupriavidus sp*. strain SHE (Qu et al., 2015a; Qu et al., 2017). Later, they isolated indole-degrading bacterium *Burkholderia sp*. IDO3 and found an *iif2* gene cluster for indole degradation and indigo production (Ma et al., 2019a; Ma et al., 2019b).

Although the indole degradation pathway has been studied for almost a century, the indole biodegradation gene cluster that can contribute to the selective Tyrian purple production has not been reported so far. In this study, we found Tyrian purple can be selectively produced in a *Providencia rettgeri* bacterial strain GS-2 from a laboratory environment. Then, we isolated and obtained the complete genomic sequence of GS-2 and identified an indole degradation gene cluster for indole metabolism in GS-2. Heterologous expression of these genes is from the indole degradation gene cluster in *Escherichia coli* BL21 (DE3). The *in vivo* and *in vitro* reaction results showed that the indole biodegradation gene cluster may contribute to the selective Tyrian purple production. Compared with the most reported monooxygenase MaFMO and cytochrome P450 enzyme CYP102G4, the Tyrian purple-producing enzyme *GS-*C co-expressed with its cofactor *GS*-D in the indole biodegradation gene cluster have the best activity in the production of Tyrian purple in *E. coli*. At last, we tried to unlock the biotransformation and degradation mechanism of selective Tyrian purple producing in GS-2. Unveiling the selective Tyrian purple production will open an avenue to promote the biosynthesis of Tyrian purple.

## 2 Materials and methods

### 2.1 Bacterial strains, chemicals, and standard techniques

*Escherichia coli* DH5α and BL21 (DE3) strains were used as cloning and protein expression hosts, respectively. *E. coli* strains carrying plasmids were cultivated in a Luria-Bertani (LB) medium supplemented with antibiotic (50 μg/mL kanamycin). An LB medium was purchased from Huankai Microbial (China). M9 media salts were purchased from Sangon Biotech (China). Plasmid DNA was isolated using a Tiangen plasmid miniprep kit (TIANGEN, Ltd. China). 6-Bromo-tryptophan and 6-chloro-tryptophan were purchased from GL Biochem (China). Tryptophan and NADH were purchased from Sigma (United States). Indole, 6-bromo-indole, indigo, FAD, and isatin were purchased from Sigma (United States), Bidepharm (China), and Yuanye Biotech (China), respectively. Indirubin was purchased from TCI (Japan). Chemically synthesized 6, 6′-dibromoindigo was synthesized from Abace Biotech (China). Gene and oligomer synthesis and sequencing were carried out in BGI (China), Sangon (China), and Rui Biotech (China), respectively. Enzymes involved in restriction reaction, ligation, and PCR were purchased from New England Biolabs (United States), Vazyme (China), and TAKARA (Japan), respectively. All other chemicals used in this study were of analytical grade. All media and reagent solutions were prepared with Milli-Q water (Merck Millipore).

### 2.2 Genome sequencing and annotation

The bacterial strain designated as GS-2 was isolated from the LB agar plates supplemented with 6-bromo-tryptophan (containing purple colonies). To obtain a partial genome sequence, total DNA from GS-2 was isolated using the TIANamp Bacteria DNA Kit (TIANGEN, China). The genome of GS-2 was sequenced with 15775578 reads of an average length of 350 bp by using an Illumina HiSeq 4000 system (Illumina, San Diego, CA, United States) at the Beijing Genomics Institute (Shenzhen, China). Raw reads of low quality from paired-end sequencing were discarded. The sequenced reads were assembled using SOAP *de novo* v1.05 software. Gene prediction was performed on the GS-2 genome assembly by Glimmer3 (http://www.cbcb.umd.edu/software/glimmer/) with hidden Markov models. tRNA, rRNA, and sRNAs recognition made use of tRNAscan-SE (Lowe and Eddy, 1997), RNAmmer, and the Rfam database. The best hit abstracted using a BLAST alignment tool for function annotation. Seven databases which are KEGG (Kyoto Encyclopedia of Genes and Genomes), COG (Cluster of Orthologous Groups), NR (non-redundant protein database), Swiss-Prot, and GO (Gene Ontology), TrEMBL, and EggNOG are used for general function annotation.

### 2.3 Identification of an indole biodegradation gene cluster

According to the reported microbial indigo-forming enzymes or indole biodegradation gene cluster and the draft genome sequencing of GS-2 and bioinformatics analysis of the genome of GS-2 using NCBI’s blast^+^ algorithm, blastn algorithm, and the Conserved Domain Database (CCD), we use the reported microbial indigo-forming enzymes or indole biodegradation gene cluster as the user’s query to the database of the GS-2’s draft genome sequences. The prevalence of indole biodegradation gene cluster as well as genes of indole oxygenase among available microbial genomes was analyzed by using the NCBI’s blast^+^ algorithm, too. Based on the blastn algorithm and the gene annotation of *Providencia heimbachae* ATCC 35613 in NCBI, seven genes of the *GS* (A-R) encoded putative enzymes, as described by conserved domain analysis.

### 2.4 Heterologous expression of indole oxygenase genes and indole biodegradation gene cluster

#### 2.4.1 Plasmid and strain construction

Genes *GS* (A-E) from genomic DNA of strain GS-2 were amplified using 2×Phanta^®^ Master Mix (Vazyme, China). The primers for amplification are provided in Table 1. The PCR products were purified and ligated with linearized pET28a (+) plasmid using a Gibson One Step Cloning Kit (New England Biolabs, United States). Tryptophanase gene (TnaA) from *E. coli* was amplified from the genomic DNA of *E. coli* BL21 (DE3) using primer TnaA_fwd and TnaA_rev, and it was cloned into pCDF, yielding pCDF:tnaA. To transform host cells with the fusion constructs, the Gibson reaction mixture (5 μL) was added to chemical competent *E. coli* DH5α cells, and a heat shock (42°C) was applied for 60 s. After overnight growth on an LB agar plate with kanamycin, colonies were picked and grown in LB, and then the plasmids were isolated and sent for sequencing (Rui Biotech, China) to confirm the correct ligation of the genes. Then, the recombinant plasmid was successfully expressed in strain *E. coli* BL21 (DE3).

**TABLE 1:** List of oligos used in this study.

Primer	Sequence
pET28a-fwd	CAC​CAC​CAC​CAC​CAC​CAC​TGA​GAT​C
pET28a-Rev	CAT​GAA​TTC​GGA​TCC​GCG​ACC​C
GS-C-fwd	GTC​GCG​GAT​CCG​AAT​TCA​TGC​GTC​GTA​TTG​CTA​TCG
GS-C-rev	CAG​TGG​TGG​TGG​TGG​TGG​TGT​TAG​GCT​CGA​GCT​TCT​TC
GS-C + D-fwd	AAC​TTT​AAG​AAG​GAG​ATA​TAC​CAT​GAT​GTC​TAT​GAT​TGC​AGA​GGA​TAC
GS-C + D-rev	CAT​CAT​GGT​ATA​TCT​CCT​TCT​TAA​AGT​TAA​TTA​GGC​TCG​AGC​TTC​TTC​CAT​C
GS-D-rev	CAG​TGG​TGG​TGG​TGG​TGG​TGC​TAA​AGG​GCT​TCA​TGT​AAA​G
GS-B-fwd	CAA​ATG​GGT​CGC​GGA​TCC​GAA​TTC​ATG​AGA​GTA​AAA​AAG​AGG​ATG​CCG​AAG
GS-B + C + D + A-rev	CAT​GGT​ATA​TCT​CCT​TCT​TAA​AGT​TAA​TTA​TTC​AGC​AAG​ACG​GGG​AAT​TG
GS-B + C + D + A-fwd	AAC​TTT​AAG​AAG​GAG​ATA​TAC​CAT​GAT​GAA​CCA​GCC​AGC​ATT​GAT​G
GS-A-rev	GAT​CTC​AGT​GGT​GGT​GGT​GGT​GGT​GTC​ACC​CCA​CCA​GCG​CTA​G
GS-O-fwd	GTC​GCG​GAT​CCG​AAT​TCA​TGA​CAA​CAC​ACT​CTC​ACC​C
GS-O-rev	CAG​TGG​TGG​TGG​TGG​TGG​TGT​TAT​GTC​CTA​CGT​TTG​AC
GS-O + E-rev	TGG​TAT​ATC​TCC​TTC​TTA​AAG​TTA​ATT​ATG​TCC​TAC​GTT​TGA​CTG​CC
GS-E-fwd	TTA​ACT​TTA​AGA​AGG​AGA​TAT​ACC​ATG​ATG​GCA​GGA​AAT​ATG​ATG​AAA​AC
GS-E-rev	GAT​CTC​AGT​GGT​GGT​GGT​GGT​GGT​GTT​AGA​ATG​GAA​TGG​CCG​C
GS-B + C + D + A + O + E-fwd	CAA​TTC​CCC​TCT​AGA​AAT​AAT​TTT​GTT​TAA​CTT​TAA​GAA​GGA​GAT​ATA​CCA​TGA​TGA​CAA​CAC​ACT​CTC​ACC​CTC​AAG
GS-B + C + D + A + O + E-rev	CAA​AAT​TAT​TTC​TAG​AGG​GGA​ATT​GTT​ATC​CGC​TCA​CAA​TTC​CCC​TAT​AGT​GAG​TCG​TAT​TAT​CAC​CCC​ACC​AGC​GCT​AG
TnaA-fwd	ATG​GAA​AAC​TTT​AAA​CAT​CTC​CC
TnaA-rev	AAC​TTC​TTT​AAG​TTT​TGC​GGT​G
MaFMO-fwd	GAA​GGA​GAT​ATA​CCA​TGA​TGG​CAA​CTC​GTA​TTG​CGA​TAC
MaFMO-rev	ATC​TCC​TTC​TTA​AAG​TTA​ATT​AAG​CTT​CTT​TAG​CCA​CAG​G
CYP102G4-fwd	TCG​AGT​GCG​GCC​GCA​AGC​TTT​CAC​CCG​GCG​GCG​TAC​AC
CYP102G4-rev	CGG​TGG​CAG​CAG​CCT​AGG​TTA​ACC​TGC​TGC​ATA​AAC​ATC

#### 2.4.2 Growth curve, growth conditions, and protein expressions

Overnight cultured GS-2 and *E. coli* were inoculated into a fresh medium, and the OD_600_ of bacteria was measured using a smart microplate reader (Infinite^®^ 200 Pro, Tecan, Switzerland) every 30 min. The experiment was repeated three times. Expression plasmids with N-terminally encoded 6×His tags were transformed into *E. coli* BL21 (DE3) for protein expression. A single colony was inoculated into an LB medium with corresponding antibiotics and grown overnight at 37°C. Then, 30 μL of the seed cultures were inoculated into 3 mL of an LB media, and they were grown at 37°C until the cell density reached an optical density (OD600) of roughly 0.4–0.6. Expressions of proteins were induced by the addition of 0.1 mM isopropyl-β-d-thiogalactoside (IPTG) with subsequent overnight incubation at 30°C. Expressions of TnaA + CYP102G4 were induced by 0.1 mM IPTG and 0.25 mM ALA (5-aminolevulinic acid) with overnight incubation at 30°C.

#### 2.4.3 Purification of proteins and enzyme assays

Expressions of *GS*-C, *GS*-D, *GS*-B, *GS*-A, GDH, and MaFMO were induced by 0.1 mM IPTG in 1 L of M9 media with overnight incubation at 18°C. The cells were collected by centrifugation at 9,000 rpm for 10 min and washed with 50 mM Tris-HCl (pH 7.5) buffer solution. The cells were resuspended in Tris buffer solution containing 50 mM Tris-HCl (pH 8.0), 500 mM NaCl, and 10% (v/v) glycerol. The cell soup was disrupted by ultrasonication in ice-chilled water for 40 min (2 s turn on and 2 s turn off). The soluble fractions were collected by centrifugation of the cell lysate at 12,000 rpm for 60 min at 4°C. The soluble fractions of the proteins were purified by Ni-NTA His-tag protein purification. The total and soluble fraction of lysates and the elution fractions were loaded into 12.5% sodium dodecyl sulfate-polyacrylamide gel electrophoresis (SDS-PAGE) at 250 V for 32 min. The elution fractions were concentrated to 3 mL using an Amicon 50 mL 10 or 30 kDa cutoff centrifugal filter. Protein concentration was determined by using the A280 feature of a Thermo Scientific 2000 Nanodrop.

The molecular weight of the purified proteins was detected by MALDI-Tof MS as follows: 2 μL of the purified protein was transferred onto an MTP 384 polished steel target (Bruker Daltonics, Billerica, MA) using a 2.5 µL pipette tip; 1 μL of α-cyano-4-hydroxycinnamic acid (CHCA) solution (acetonitrile: H_2_O: trifluoroacetic acid (TFA) = 50:47.5:2.5, v/v) was then spotted. MALDI-ToF mass spectra were acquired using a Bruker Autoflex MALDI-ToF/ToF mass spectrometer (Bruker Daltonics) equipped with a frequency tripled Nd:YAG solid state laser (*λ* = 355 nm). Mass spectrometer calibration was performed using Peptide Calibration Standard Kit II (Bruker Daltonics). Spectral acquisition was performed in positive reflection mode with pulsed ion extraction and a mass range of 10,000–70,000 Da. The laser footprint was set to “Ultra” at a ∼100 µm diameter, and 500–1,000 laser shots were fired at 1,000 Hz. Mass spectra were smoothed, baseline-corrected, and analyzed in FlexAnalysis 3 (Bruker Daltonics).

#### 2.4.4 *In vivo* reactions

For *in vivo* substrate reaction, 1 mM tryptophan or 1 mM 6-bromo-tryptophan, 1 mM indole, or 0.25 mM 6-bromo-indole were added into the overnight induced culture, respectively. For the identification of the monooxygenase selectivity, feeding 1 mM tryptophan and 1 mM 6-bromo-tryptophan or 1 mM indole and 0.25 mM 6-bromo-indole simultaneously. Indigo/Tyrian purple concentration was determined by LC-MS analysis described as follows.

#### 2.4.5 Enzyme kinetics and *in vitro* reactions

For the enzyme kinetics reaction, indole oxidation activity of *GS*-C and *GS*-D were determined as a function of indigo/Tyrian purple formation in the reaction mixture, respectively. Initial indole oxidation rather than spontaneous dimerization was assumed to be a rate-limiting step. A typical reaction mixture contained 50 mM Tris-HCl, pH 8.0, 200 μM FAD, 250 μM NADH, and various concentrations of indole (10–1,000 μM) or 6-Br-indole (10–1,000 μM) and 300 μg of *GS*-C as well as 300 μg of *GS*-D were used for the analysis of the enzyme kinetics. A typical reaction mixture contained 50 mM Tris-HCl, pH 7.5, 250 μM NADH, 0.6% glucose, and various concentrations of indole (10–1,000 μM) or 6-Br-indole (10–1,000 μM) and 300 μg of MaFMO as well as 5 U/mL of GDH were used to analyze the enzyme kinetics. Reaction mixtures were incubated at 30°C for 120–240 min. One unit of enzyme activity was defined as the amount catalyzing the formation of 1 μmol of indigo/Tyrian purple per minute.

Flavin reductase activity of the purified *GS*-D protein was determined from the decrease of the absorbance at 340 nm due to the oxidation of NADH, using a microplate reader and was performed at 30°C. A total reaction volume of 0.2 mL contained 50 mM Tris-HCl, pH 8.0, 250 μM NADH, and 100 μM FAD. The reactions were initiated by adding 300 μg of *GS*-D. One unit (U) of enzyme activity was defined as the amount of enzyme catalyzing the oxidation of 1 μmol of NADH per minute.

For *in vitro* substrate reaction, indole oxidation activity of *GS*-A, *GS*-B, *GS*-C, and *GS*-D were determined as a function of indigo/Tyrian purple formation in the reaction mixture, respectively. A typical reaction mixture contained 50 mM Tris-HCl, pH 8.0, 200 μM FAD, 250 μM NADH, and 1,000 μM indole or 1,000 μM 6-Br-indole and 100 μg of *GS* proteins were used for the *in vitro* substrate reaction. Reaction mixtures were incubated at 30°C for 150 min. Indigo/Tyrian purple concentration was determined by LC-MS analysis described as follows.

### 2.5 Identification of metabolite

For qualitative and quantitative analyses of indigo/Tyrian purple, 0.1 mL of the reaction mixtures were centrifuged at 12,000 rpm, 10 min, and the supernatant was removed. The pellet was suspended in 1 mL of DMSO followed by vigorous vortexing. The mixtures were centrifuged at 12,000 rpm, 10 min, and the supernatant was filtered through 0.22 μm filters for LC-MS analysis. For LC-MS analysis of the solid plate of *E. coli* strain expressing different genes by *in vivo* reactions, the pellet was resuspended in 1 mL DMSO and the supernatant was sent for LC-MS analysis after centrifugation. LC connected with tandem mass spectrometry (Agilent 6470 QQQ) using the C18 reversed-phase (4.6 × 100 mm, 2.7-Micron). LC-MS data were collected using the Agilent MassHunter Workstation. The condition for LC gradient wash was as follows: a gradient of H_2_O+ 10 mM ammonium acetate (solvent A) and acetonitrile (solvent B) using the following method: 60%–50% B for 16–40 min, at a flow rate of 0.5 mL min^−1^. The mass spectrometry conditions were as follows: column temperature 40°C; electrospray ionization in negative mode; capillary voltage 3.8 kV; vaporizer temperature 350°C; capillary temperature 320°C; sheath gas pressure 30 psi; auxiliary gas pressure 10 psi; and SIM event 2, mass for the analysis of indigo and indirubin is 261. Mass for the analysis of 6, 6′-dibromoindigo is Tyrian purple, TP; m/z 417, 419, and 421 and 6, 6′-dibromoindirubin is TP isomer; m/z 417, 419, and 421.

For liquid chromatography electrospray ionization quadrupole time-of-flight mass spectrometry (LC-ESI-QTOF-MS/MS) analysis, the sample extraction was as follows: 0.2 mL of the sample from each reaction was collected in a new 1.5 mL centrifuge tube and freeze dried for 3–5 h. The solid was then dissolved in 1 mL ethanol with 0.1% formic acid and treated with ultrasound for 1.5 h. Each sample was centrifuged (12,000 rpm, 10 min) to take the supernatant, which was filtered through 0.22 μm filters for analysis. The condition for LC gradient wash was as follows: a gradient of H_2_O+ 0.1% formic acid (solvent A) and acetonitrile +0.1% formic acid (solvent B) using the following method: 2% B for 1 min, 2%–40% B for 4 min, 40%–70% B for 5 min, 70%–95% B for 3 min, and 95% B for 4 min and re-equilibration 2% B for 3 min. A volume of 5 µL was injected for each standard or sample and the flow rate was set at 0.3 mL/min. Nitrogen gas nebulization was set at 45 psi with a flow rate of 10 L/min at 350°C, and the sheath gas was set at 12 L/min at 350°C. The capillary and nozzle voltage were set at 4 kV and 1,500 V, respectively. A complete mass scan ranging from m/z 50 to 1,300 was used, and MS/MS analyses were carried out in automatic mode with collision energy (10, 20, and 40 eV) for fragmentation. Peak identification was performed in both positive and negative modes while the instrument control, data acquisition, and processing were performed using MassHunter Workstation software (Qualitative Analysis, version 10.0) (Agilent Technologies, Santa Clara, CA, United States).

### 2.6 Indole, 6-Br-indole, Tyrian purple, indirubin, and indigo toxicity test

To test the effects of different concentrations of indole, 6-Br-indole, Tyrian purple, indirubin, and indigo on the growth of *E. coli* BL21 (DE3), 3 mL of overnight LB culture media was collected and centrifuged. The supernatant was discarded, and the pellet was resuspended in 1 mL of LB. This aliquot was then transferred to a flask containing 70 mL of LB and incubated. Thus, after adding varying amounts of indole, 6-Br-indole (dissolved in absolute ethanol), Tyrian purple, indirubin, and indigo (dissolved in dimethylsulfoxide), the culture was incubated at 37°C, 220 rpm for 17 h. The optical density at 600 nm (OD600) was used to determine the growth of the bacteria under the various conditions. The experiments were performed in triplicate.

## 3 Results

### 3.1 Tyrian purple can be selectively produced in a bacterial strain

**FIGURE 2:**
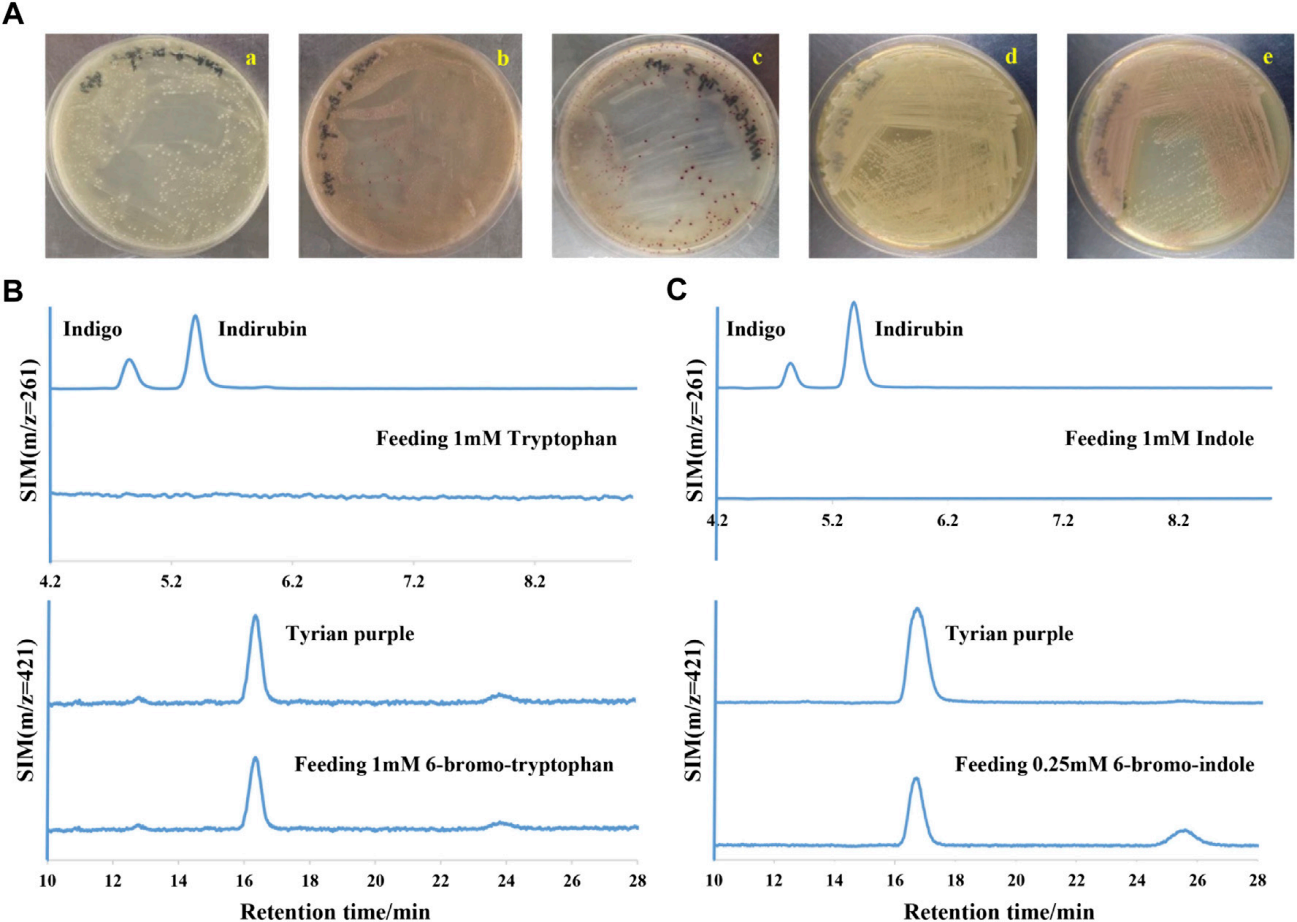
Tyrian purple can be selectively produced in GS-2. **(A)** GS-2 grown on the LB agar plates supplemented with different substrates: a. 1 mM Tryptophan; b. 1 mM 6-chloro-tryptophan; c. 1 mM 6-bromo-tryptophan; d. 1 mM Indole; and e. 0.25 mM 6-bromo-indole. **(B)** Extracted ion chromatograms for standards (the upper) of indigo (m/z = 261), indirubin (m/z = 261), Tyrian purple (m/z = 421), and colonies (the lower) were collected from GS-2 grown on the LB agar plates supplemented with tryptophan or 6-bromo-tryptophan, respectively. **(C)** Extracted ion chromatograms for the standards (the upper) of indigo (m/z = 261), indirubin (m/z = 261), Tyrian purple (m/z = 421), and colonies (the lower) were collected from GS-2 grown on the LB agar plates supplemented with indole or 6-bromo-indole, respectively.

In a laboratory environment, we accidentally found some purple colonies as contaminants on Luria-Bertani (LB) agar plates supplemented with 1 mM 6-bromo-tryptophan (6-Br-Trp) or 1 mM 6-chloro-tryptophan (6-Cl-Trp) (Figure 2A). Based on the previous study on biological synthesis of 6, 6′-dibromoindigo (Tyrian purple) from tryptophan (Trp), 6-Br-Trp is an intermediate in the Tyrian purple biosynthesis pathway (Lee et al., 2021). Whether these purple colonies were caused by the presence of Tyrian purple? To confirm our hypothesis, the purple colonies were collected and dissolved into dimethylsulfoxide (DMSO) and analyzed by high-performance liquid chromatography mass spectrometry (LC-MS). The results showed that the purple pigments were Tyrian purple (Figure 2B). To validate the Tyrian purple biosynthesis pathway from 6-Br-Trp, 6-bromo-indole (6-Br-indole) was evaluated through substrate reaction. Notably, this strain produced Tyrian purple when grown on an LB agar plate supplemented with 0.25 mM 6-Br-indole (Figure 2C). Halo-tryptophan is tryptophan analogs, if a strain can utilize halo-tryptophan to produce purple pigments, it should also mediate the indigo and indirubin production from tryptophan (Namgung et al., 2019; Schnepel et al., 2021) (Figure 1). However, we verified there was neither indigo nor indirubin production in the colonies from LB agar plates supplemented with the tryptophan or indole (Figure 2). According to the results, we supposed this strain can produce Tyrian purple selectively from Trp/6-X-Trp (X = Cl, Br) or indole/6-X- indole (X = Cl, Br).

### 3.2 Characterization of strain GS-2

The growth curve of GS-2 based on OD is shown in Supplementary Figure 1A. The curve shows that GS-2 reached the stationary phase at about 10 h. To further explore the underlying mechanism for the selectivity, the bacterial strain found to be a selectively Tyrian purple producer was isolated and designated as GS-2. The bacterial genome sequencing analysis predicted that GS-2 is *Providencia rettgeri* with tax number 587. The genome size of GS-2 is 4,874,999 bp. The 4,658,124 bp obtained reads were *de novo* assembled into 79 scaffolds, with a 95.55% of the assembly genome of the sequenced strain (Table 2). The alignment length is 4,266,344 bp, with a 91.59% of the scaffold length. The genome of strain GS-2 had an overall GC content of 41.52%. A total of 4,565 genes, including 137 RNAs, were predicted in the genome. The genome sequencing result of GS-2 have been successfully submitted to NCBI (SRA accession number: JAPQLO000000000), and the genomic information of strain GS-2 facilitates the molecular mechanism and bioremediation study of *Providencia rettgeri*.

**TABLE 2:** Bacteria genome sequencing result of *Providencia rettgeri* GS-2.

Sample name	Tax ID	Organism	Cover_Len (bp)	Scaffolds_Len	Coverage (%)	Genomics (%)	Scaffold_N
Mut.GS-2	587	*Providencia rettgeri*	4266344	4658124	91.59	95.55	50
	546	*Citrobacter freundii*	153186	153186	100.00	3.14	1
	1157951	*Providencia stuartii*	16099	24891	64.68	0.51	2
	6239	*Caenorhabditis elegans*	13263	13275	99.91	0.27	11
	333962	*Providencia heimbachae*	5473	12461	43.92	0.26	2

Tax ID: species tax id, Organism: species name, Cover_aLen: Alignment coverage length, Scaffolds_Len: alignment to the length of the sequenced strain scaffold on this species, Coverage: alignment length as a percentage of the scaffold length, Genomics: scaffold length as a percentage of the sequence length of the assembly genome of the sequenced strain, Scaffold_Num: the number of scaffolds aligned to the species.

*Providencia* is a ubiquitous Gram-negative bacterium in the family of *Enterobacteriaceae*, which are considered as opportunistic pathogens (Galac and Lazzaro, 2012; Sadauskas et al., 2017). *Providencia rettgeri* is an indole-positive bacterium, which can produce indole by TnaA from l **-**tryptophan. According to several reports on the biological production of indigo itself in *E. coli*, various colored indigoid dyes can be generated by feeding appropriate halogenated indoles as a substrate to oxygenases, naphthalene dioxygenase, and toluene dioxygenase (Qu et al., 2012; Zhang et al., 2013; Fukuoka et al., 2015; Heine et al., 2019; Choi, 2020; Mendoza-Avila et al., 2020; Lee et al., 2021). When the GS-2 strain was grown on LB agar plates supplemented with Trp, 6-Br-Trp or indole, and 6-Br-indole, Tyrian purple was the only indigoid product (Figure 2). These results indicated that there may exist a selective oxygenase (only oxidize halogenated indole) in strain GS-2. Otherwise, there may be a selective indole biodegradation gene cluster (degrade the indole, but not 6-Br-indole) in strain GS-2.

### 3.3 Bioinformatics analysis of the genes involved in indole degradation and oxidation

Bioinformatics analysis of the genome of GS-2 by the NCBI’s blast^+^ algorithm with the reported microbial indigo-forming enzymes or indole biodegradation gene cluster allowed the identification of a set of genes (here designated *GS* (A-R)) (Table 3) for indole degradation and oxidation (Ma et al., 2018; Fabara and Fraaije, 2020). These genes *GS* (A-R), which were located in a 7-kb genomic fragment of GS-2, were further analyzed based on the NCBI’s blastn algorithm and the Conserved Domain Database (CCD) with other *Providencia* species. According to the prediction of *Providencia heimbachae* ATCC 35613, these genes *GS* (A-R) involved in the indole degradation and oxidation were described as the conserved domain predication, such as *GS-*A encodes a cyclase family protein, arylformamidase activity. *GS-*B encodes a short-chain dehydrogenase/reductase and oxidoreductase activity. *GS-*C encodes a styrene monooxygenase. *GS-*D encodes a flavin reductase, FMN binding, and monooxygenase activity. *GS-*E is a putative MetA-pathway of phenol degradation. *GS-*O encodes oxidoreductase. In addition, a putative AraC transcription regulator, *GS-*R occurred.

**TABLE 3:** Predicted conserved domains and putative functions of proteins encoded in *Providencia rettgeri* GS-2 according the prediction of *Providencia heimbachae* ATCC 35613.

Protein	Gene_id	Length/bp	Identity (%)	E_value	Accession_id	Function and conserved domain
*GS*-A	Mut.GS.2GL000526	782	90	0	A0A1B7JR90	Arylformamidase activity, cyclase (PF04199)
*GS*-B	Mut.GS.2GL000529	785	92	9.3E^−170^	A0A1B7JRB6	Short-chain dehydrogenase/reductase, oxidoreductase activity
*GS*-C	Mut.GS.2GL000528	1244	94	0	A0A1B7JR97	Styrene monooxygenase A putative substrate binding domain
*GS*-D	Mut.GS.2GL000527	530	89	6.9E^−115^	A0A1B7JR95	Flavin_Reduct (PF01613), FMN binding, and monooxygenase activity
*GS*-E	Mut.GS.2GL000524	908	91	0	A0A1B7JRB5	Putative MetA-pathway of phenol degradation (PF13557)
*GS*-R	Mut.GS.2GL000523	899	94	0	A0A1B7JR83	AraC family transcriptional regulator
*GS*-O	Mut.GS.2GL000525	1613	90	0	A0A1B7JR88	Oxidoreductase activity (GO:0016491)

**FIGURE 3:**
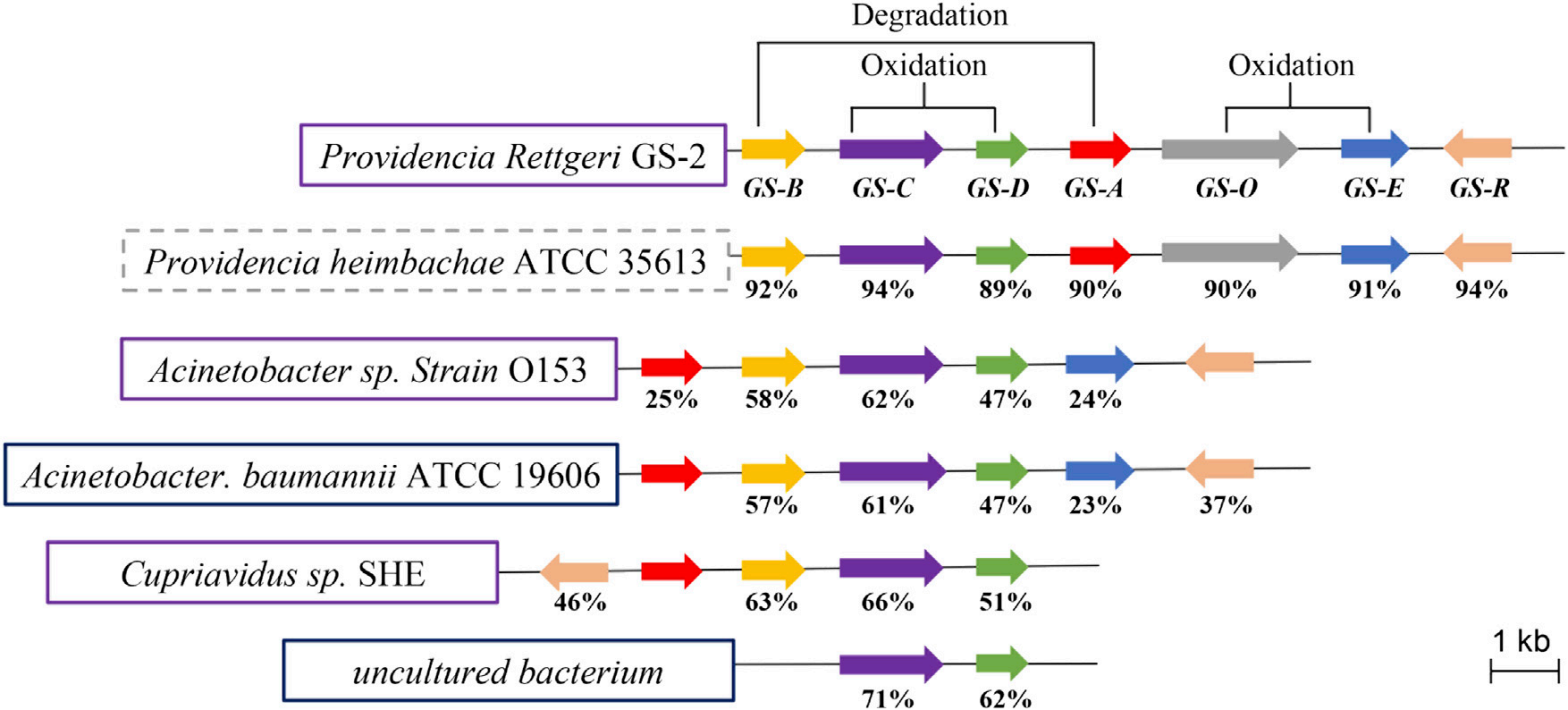
Organization and distribution of GS (A-R) genes in different microbial genomes. Genes are represented by arrows (drawn to scale as indicated). Homologous genes are highlighted in the same pattern according to the scheme in the top line for GS-2. Strains and genomic fragments boxed in solid lines indicate reported activity of certain GS-homologous proteins; dashed boxes highlight strains for which indole biodegradation activity was reported at the gene level. Identities (percent) of amino acid sequences between GS proteins of strain GS-2 and homologs are indicated under the corresponding ORFs.

To further predict the functions of *GS* (A-R) genes, we analyzed the prevalence of *GS* (A–R) and indigo-forming genes among available microbial genomes (Figure 3). Comparing with those species (*Acinetobacter*, *A. baumannii* and *Cupriavidus*) have the ability of indole degradation and indigo formation (Lin et al., 2012; Sadauskas et al., 2017; Ma et al., 2019a; Ma et al., 2019b), the *GS*-C and *GS*-D genes were found to be a common component among different microbials with high similarity. From previous research, *GS-*C homologues genes were identified as indigo-forming enzymes (monooxygenase activity was detected by oxidizing indole to indigo) in many microbials (O'Connor et al., 1997; Drewlo et al., 2001; Doukyu et al., 2003; Alemayehu et al., 2004; Lu and Mei, 2007; Kwon et al., 2008; Ameria et al., 2015; Qu et al., 2015b; Heine et al., 2019; Fabara and Fraaije, 2020). The *GS*-D gene encoded a flavin reductase, flavin adenine dinucleotide (FAD) cofactor, and NAD(P)H, which supposed that *GS*-C was possibly a cofactor-independent oxygenase that catalyzed the transformation of indole to indigo. *GS-*A and *GS-*B genes were involved in the indole degradation along with *GS-*C and *GS-*D genes. It is worth noting that *GS-*O encodes oxidoreductase appears only in *Providencia*, and the function of *GS-*O has not been identified. Therefore, *GS-*O may be a selective oxygenase in strain GS-2.

### 3.4 Characterization of the *GS* proteins in *E. coli*

**FIGURE 4:**
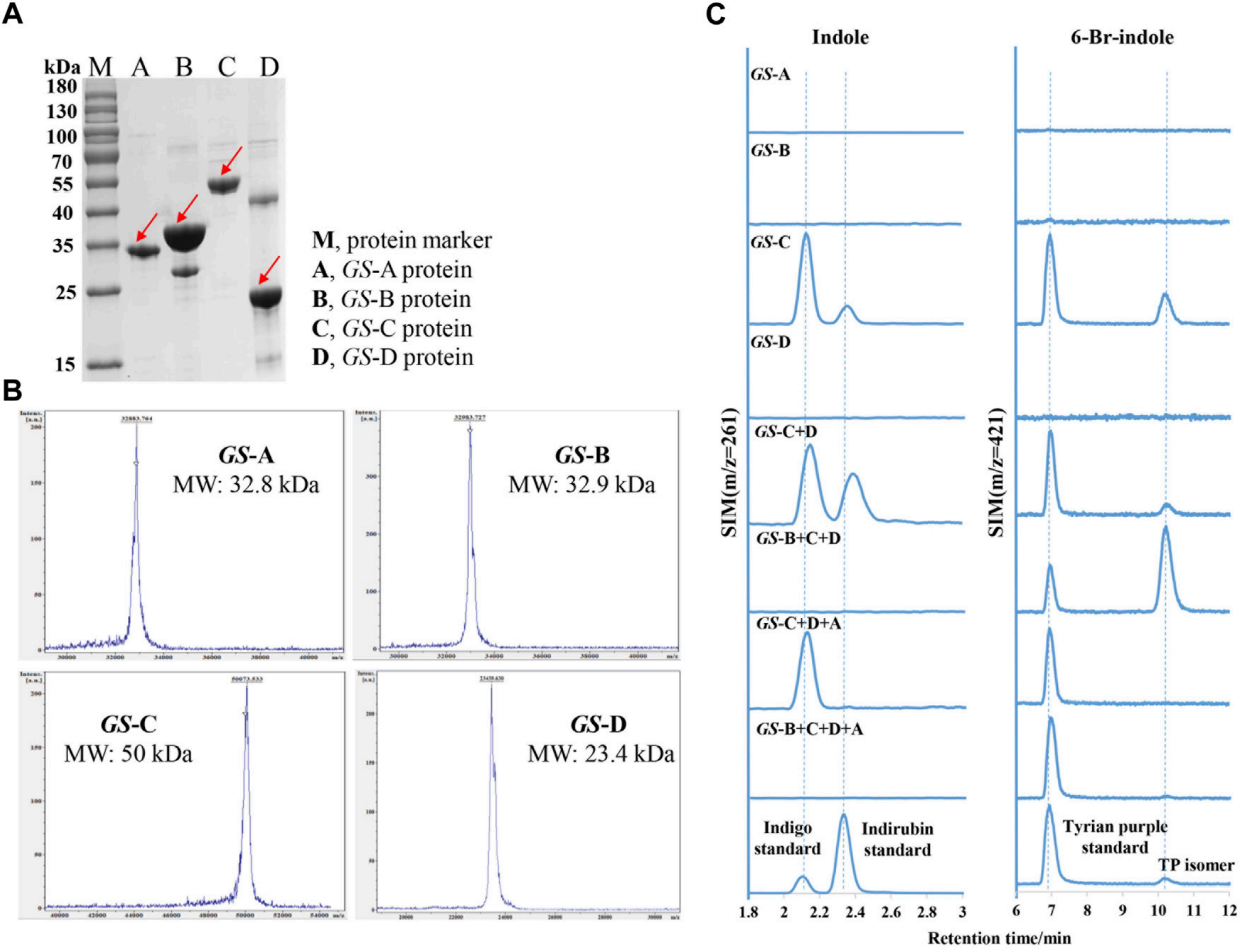
Purification and characterization of indole biodegradation gene cluster. **(A)** SDS-PAGE analysis of the purified four *GS* proteins. M, protein marker; A, *GS*-A; B, *GS*-B; C, *GS*-C; D, *GS*-D. **(B)** MALDI-TOF mass spectrometry of the purified four *GS* proteins. **(C)** Extracted ion chromatograms for the standards (the lower) of indigo (m/z = 261), indirubin (m/z = 261), Tyrian purple (m/z = 421), and production of *in vitro* reactions by different *GS* proteins supplemented with indole or 6-bromo-indole, respectively.

To elucidate the functions of these *GS* proteins, *GS-*A, *GS-*B, *GS-*C, *GS-*D, *GS-*O, and *GS-*E genes were successfully overexpressed in *E. coli* BL21 (DE3) (which possesses TnaA), with an N-terminal His-tag, *GS-*A, *GS-*B, *GS-*C, and *GS-*D were successfully purified, and their activity toward Trp, 6-Br-Trp, indole, and 6-Br-indole was tested, which were monitored by LC-MS. Molecular masses of the purified proteins observed in SDS-PAGE gels and MALDI-TOF mass spectrometry corresponded well to the theoretical masses (32.8 kDa for *GS-*A, 32.9 kDa for *GS-*B, 50 kDa for *GS-*C, and 23.4 kDa for *GS-*D) (Figure 4 A and B).

**FIGURE 5:**
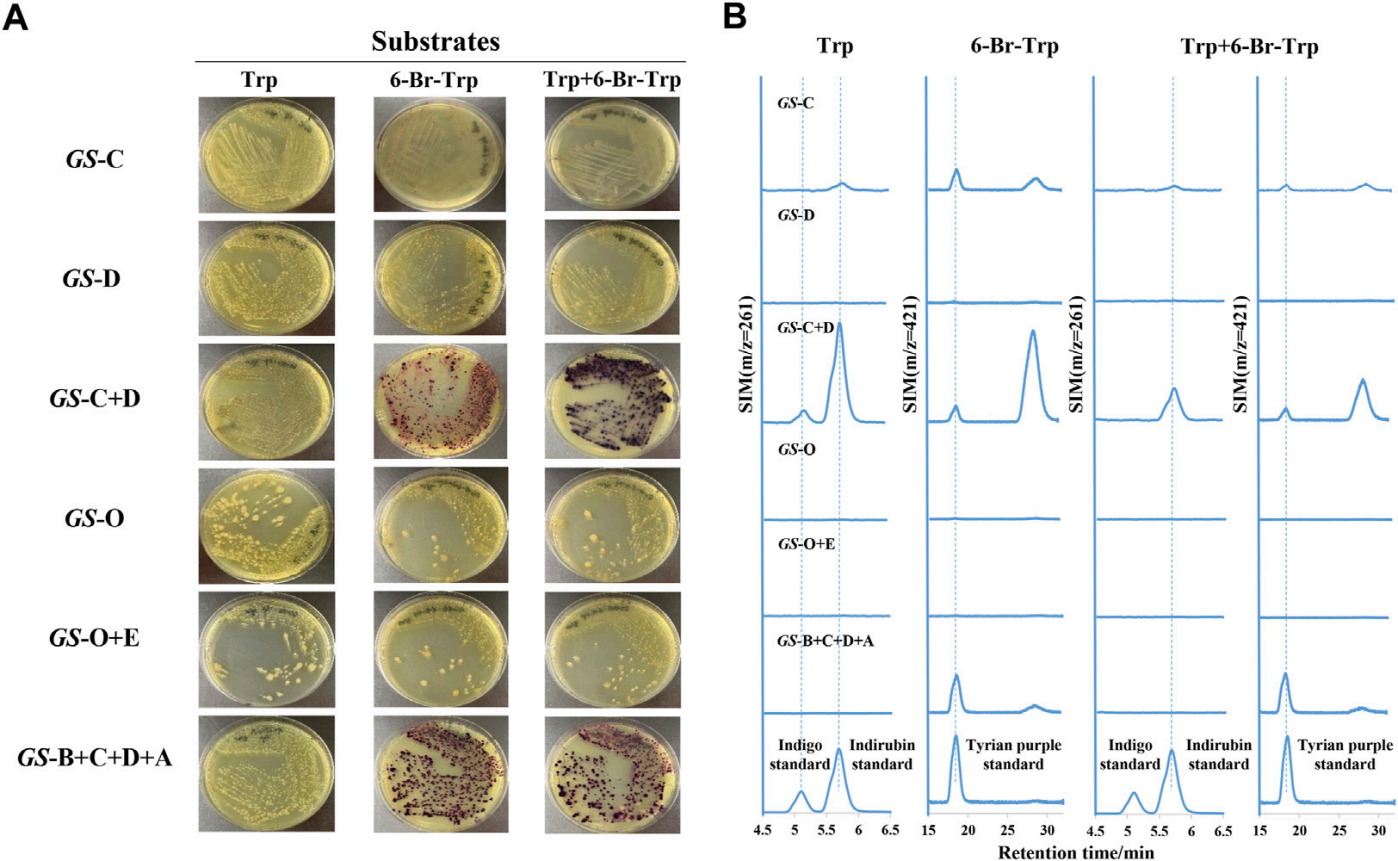
Heterologous expression of indole oxygenase genes and indole biodegradation gene cluster in *E. coli* BL21 (DE3). **(A)** Engineered *E. coli* strains with different *GS* proteins were respectively grown on agar plates with tryptophan and 6-bromo-tryptophan. *GS*-C, heterologous expression of *GS*-C; *GS*-D, heterologous expression of *GS*-D; *GS*-C + D, heterologous expression of *GS*-C and *GS*-D; *GS*-O, heterologous expression of *GS*-O; *GS*-O + E, heterologous expression of *GS*-O and *GS*-E; *GS-B + C + D + A*, and heterologous expression of *GS*-B, *GS*-C, *GS*-D, and *GS*-A. **(B)** Extracted ion chromatograms for the standards (the lower) of indigo (m/z = 261), indirubin (m/z = 261), Tyrian purple (m/z = 421), and colonies were collected from *E. coli* strains expressing different *GS* proteins on the LB agar plates supplemented with tryptophan and 6-bromo-tryptophan.

Bioinformatics analysis of the *GS*-C gene predicted that *GS*-C was possibly an indigo-forming enzyme, so its activities toward indole and 6-Br-indole were evaluated by *in vitro* reactions first. When 1 mM indole was used as the substrate, *GS*-C produced indigo and indirubin (Figure 4C). When 1 mM 6-Br-indole was used as a substrate, Tyrian purple and TP isomer were produced. Though *GS*-C was found to be capable of using indole or 6-Br-indole as substrates, indole and 6-Br-indole were toxic to the growth of *E. coli* (Lin et al., 2015), so Trp and 6-Br-Trp were added to the culture of *GS*-C in *E. coli* BL21 (DE3) (which possesses TnaA, Trp and 6-Br-Trp can be catalyzed to indole and 6-Br-indole, respectively) instead. The transformed product of *GS*-C in *E. coli* BL21 (DE3) supplied with 1 mM Trp substrate was a blue insoluble indigoid pigment, which was identified as indigo and indirubin by LC-MS (Figure 5). The strain supplemented with 1 mM 6-Br-Trp showed the purple pigment, which was identified as Tyrian purple and TP isomer by LC-MS. When feeding 1 mM Trp and 1 mM 6-Br-Trp simultaneously, the transformed product of which was a blue–purple insoluble indigoid pigment (Figure 5A). Hence, we suggested that *GS*-C was functional as oxygenase for Tyrian purple biosynthesis without observable selectivity toward indole or 6-Br-indole.

The *in vitro* reaction results of *GS-*D showed that there was neither Tyrian purple nor indigo/indirubin, and TP isomer be detected by LC-MS when 1 mM indole or 1 mM 6-Br-indole was used as substrate. Based on the properties of two-component FAD-dependent indole monooxygenases, both protein components are usually encoded next to each other on the genome in a respective gene cluster (Heine et al., 2018). Since *GS*-D was proposed as a cofactor generating enzyme and located near *GS*-C in the genome, we were wondering whether the addition of *GS*-D to *GS*-C strain can improve the activity of *GS*-C, and the *in vivo* reaction results showed that with *GS*-D added, there were more blue/purple pigments than which produced by *GS*-C in *E. coli* (Figure 5A). It is easy to distinguish the differences between *GS*-C and *GS*-C + D by the results of ion chromatograms (Figure 5B). We also characterized the flavin reductase activity of the purified *GS*-D reductase. As shown in Supplementary Figure 2, the decrease of the absorbance at 340 nm due to the oxidation of NADH demonstrated the flavin reductase activity of the *GS*-D reductase. The catalytic results of *GS-*C and *GS-*D are consistent with the homologous enzymes reported previously (Dai et al., 2019; Heine et al., 2019).

To identify whether the *GS-*O is a selectivity oxygenase in the strain GS-2, *GS-*O was expressed in *E. coli* BL21 (DE3) and cultured with Trp and 6-Br-Trp, there was no colony with any observable blue or purple color on the plates (Figure 5A), and none of Tyrian purple, indigo/indirubin, and TP isomer was identified by LC-MS (Figure 5B). These results indicated that the *GS*-O may not work as the Tyrian purple-producing oxygenase in strain GS-2. To further verify the hypotheses that *GS-*O may be capable of using indole as a substrate only in the presence of *GS-*E, *GS-*O and *GS-*E were co-expressed in *E. coli* BL21 (DE3) cultured with Trp and 6-Br-Trp, neither blue pigment nor purple pigment occurred, too (Figure 5A). These results indicated that *GS-*O and *GS-*E were not the selective Tyrian purple-producing oxygenase in strain GS-2.

To identify whether the indole degradation gene cluster is involved in the selective Tyrian purple production in strain GS-2, we added *GS*-B to the reaction mixture (*GS*-C, *GS*-D, FAD, NADH, and indole), the indigo formation was abolished, and TP isomer formation increased. When *GS*-A was added to the reaction mixture (*GS*-C, *GS*-D, FAD, NADH, and 6-Br-indole), TP isomer formation was abolished. When the four *GS* proteins (*GS*-A, *GS*-B, *GS*-C, and *GS*-D) were used in a reaction with indole or 6-Br-indole, Tyrian purple was the only target product. In addition, we co-expressed the genes *GS-*A, *GS-*B with *GS-*C, and *GS-*D in *E. coli* BL21 (DE3) supplemented with Trp and 6-Br-Trp. The recombinant strain produced a lot of yellow colonies on the plate cultured with 1 mM Trp. There were purple insoluble indigoid pigments on the plate cultured with 1 mM 6-Br-Trp. Purple pigment was observed when feeding 1 mM Trp and 1 mM 6-Br-Trp simultaneously (Figure 5A). LC-MS identified the purple pigment as Tyrian purple, and the common indole transformation products, indigo and indirubin were not detected (Figure 5B). These *in vivo* and *in vitro* reaction results showed that the genes *GS-*A and *GS-*B may relate to the indole degradation, and the four *GS* proteins (*GS-*A, *GS-*B, *GS-*C, and *GS-*D) gene cluster may contribute to the selective Tyrian purple production. Based on the results presented before, *GS*-C was the oxygenase for Tyrian purple biosynthesis without observable selectivity toward indole or 6-Br-indole, and *GS-*D was a cofactor-generating enzyme of *GS*-C. The indole biodegradation pathway containing the four *GS* proteins (*GS*-A, *GS*-B, *GS*-C, and *GS*-D) may contribute to the selective Tyrian purple production.

### 3.5 Indole biodegradation pathway

To further investigate the molecular mechanisms underpinning this selective Tyrian purple-producing performance, we identified the metabolites involved in the indole biodegradation pathway. A qualitative analysis of the intermediates from indole-degraded extracts was achieved by using liquid chromatography electrospray ionization quadrupole time-of-flight mass spectrometry (LC-ESI-QTOF-MS/MS) analysis. Several indole degradation bacteria have been isolated and characterized for aerobic biodegradation of indole (Lin et al., 2012; Qu et al., 2015a; Sadauskas et al., 2017; Qu et al., 2017; Ma et al., 2018; Zhang et al., 2018; Ma et al., 2019a). Three major pathways for indole mineralization have been proposed, and these pathways are the catechol pathway, gentisate pathway, and anthranilate pathway (Arora et al., 2015). The proposed compounds related to the indole degradation were tentatively identified from their m/z value and MS spectra in both negative and positive ionization modes ([M − H]^−^/[M + H]^+^) using Agilent LC-MS Qualitative Software and Personal Compound Database and Library (PCDL). Compounds with mass error < ± 5 ppm and PCDL library score more than 80 were selected for further MS/MS identification and m/z characterization purposes (Zhong et al., 2020). As shown in Table 4, the metabolite of isatin was identified according to the comparison with the standard: isatin (Rt = 5.242 min [M-H]^−^ m/z = 146.0244). Additionally, we identified N-formylanthranilic acid (Rt = 3.599 min [M-H]^−^ m/z = 164.0355). 2,3-Dihydroxyindole (Rt = 2.213 min [M + H]^+^ *m*/z = 150.0555), anthranilic acid (Rt = 3.152 min [M-H]^−^ m/z = 136.0406), salicylic acid (Rt = 4.623 min [M-H]^−^ m/z = 137.0247), and catechol (Rt = 5.049 min [M-H]^−^ m/z = 109.0295). Moreover, we observed little response in indoxyl (Rt = 5.389 min; m/z = 133.0529 [M + Na]^+^ m/z = 156.0431), and indoxyl is a transient product, which is difficult to identify, so further investigation will be needed to identify its chemical structures.

**TABLE 4:** Results of all product ion scan analyses of biotransformation products related to indole degradation by LC-ESI-QTOF-MS/MS.

Proposed products	Formula	RT/min	Ionization (ESI^+^/ESI^−^)	Molecular weight	Theoretical (m/z)	Observed (m/z)	Score	Mass error (ppm)	MS/MS product ions
Indoxyl	**C**_**8**_ **H**_**7**_ **NO**	**1.130**		**133.0522**		**156.0413**	**75.07**	**-4.77**	**156.0431 and 56.0497**
2,3-Dihydroxyindole	**C**_**8**_ **H**_**7**_ **NO**_**2**_	**2.123**	**[M + H]** ^**+**^	**149.0471**	**150.0549**	**150.0555**	**97.7**	**3.64**	**132.0447, 122.0626, 104.0498, 94.0640, 77.0387, and 56.0487**
Isatin	**C**_**8**_ **H**_**5**_ **NO**_**2**_	**4.047**	**[M − H]** ^**−**^	**147.031**	**146.0236**	**146.0257**	**83.14**	**7.54**	**118.0301 and 41.9986**
N-formylanthranilic acid	**C**_**8**_ **H**_**7**_ **NO**_**3**_	**3.599**	**[M − H]** ^**−**^	**165.0420**	**164.0342**	**164.0355**	**99.49**	**1.43**	**92.0506 and 120.0455**
Anthranilic acid	**C**_**7**_ **H**_**7**_ **NO**_**2**_	**3.599**	**[M − H]** ^**−**^	**137.0471**	**136.0393**	**136.0406**	**86.55**	**1.67**	**92.0502 and 42.0357**
Salicylic acid	**C**_**7**_ **H**_**6**_ **O**_**3**_	**4.623**	**[M − H]** ^**−**^	**138.0311**	**137.0233**	**137.0247**	**98.65**	**1.84**	**93.0354, 65.0393, and 50.0035**
Catechol	**C**_**6**_ **H**_**6**_ **O**_**2**_	**5.049**		**110.0362**	**109.0284**	**109.0295**	**85.98**	**0.19**	

* Proposed products were detected in both negative [M−H]^−^ and positive [M + H]^+^ mode of ionization. RT = stands for “retention time”. Theoretical (m/z) stands for the theoretical m/z under certain mode of ionization. Observed (m/z) stands for the detected m/z. Mass error stands for the difference between theoretical m/z and observed m/z in ppm.

**FIGURE 6:**
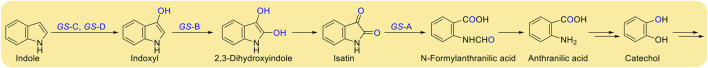
Proposed indole biodegradation pathway in *Providencia rettgeri* GS-2.

The identified degradation products after indole was completely degraded are consistent with the reported catechol pathway (Arora et al., 2015), so we proposed the indole biodegradation pathway in *Providencia rettgeri* GS-2 is the catechol pathway (Figure 6). Degradation starts with indole oxidation by *GS-*CD at the C-2 and C-3 positions, forming indoxyl. This compound is known to be rather unstable and therefore was not detected. Indoxyl is prone to auto-oxidation and forms an insoluble indigo pigment. However, the auto-oxidation could be prevented by *GS-*B, the hypothetical short-chain dehydrogenase. *GS-*B performs oxidation at the C-2 position to obtain 2, 3-dihydroxyindole. 2, 3-Dihydroxyindole is spontaneous to forming a stable isatin intermediate. N-formylanthranilic acid, the yellow product, which is catalyzed by *GS-*A, is then further degraded to anthranilic acid. Anthranilic acid was ultimately generated as the typical downstream product, which would be further degraded *via* salicylic acid and catechol. As it stands, the proposed indole biodegradation pathway in *Providencia rettgeri* GS-2 is the catechol pathway that requires additional experiments to prove the role of putative catalytic by *GS* proteins (*GS*-A, *GS*-B, *GS*-C, and *GS*-D), and these experiments are underway.

### 3.6 Production of Tyrian purple in *E. coli*

**FIGURE 7:**
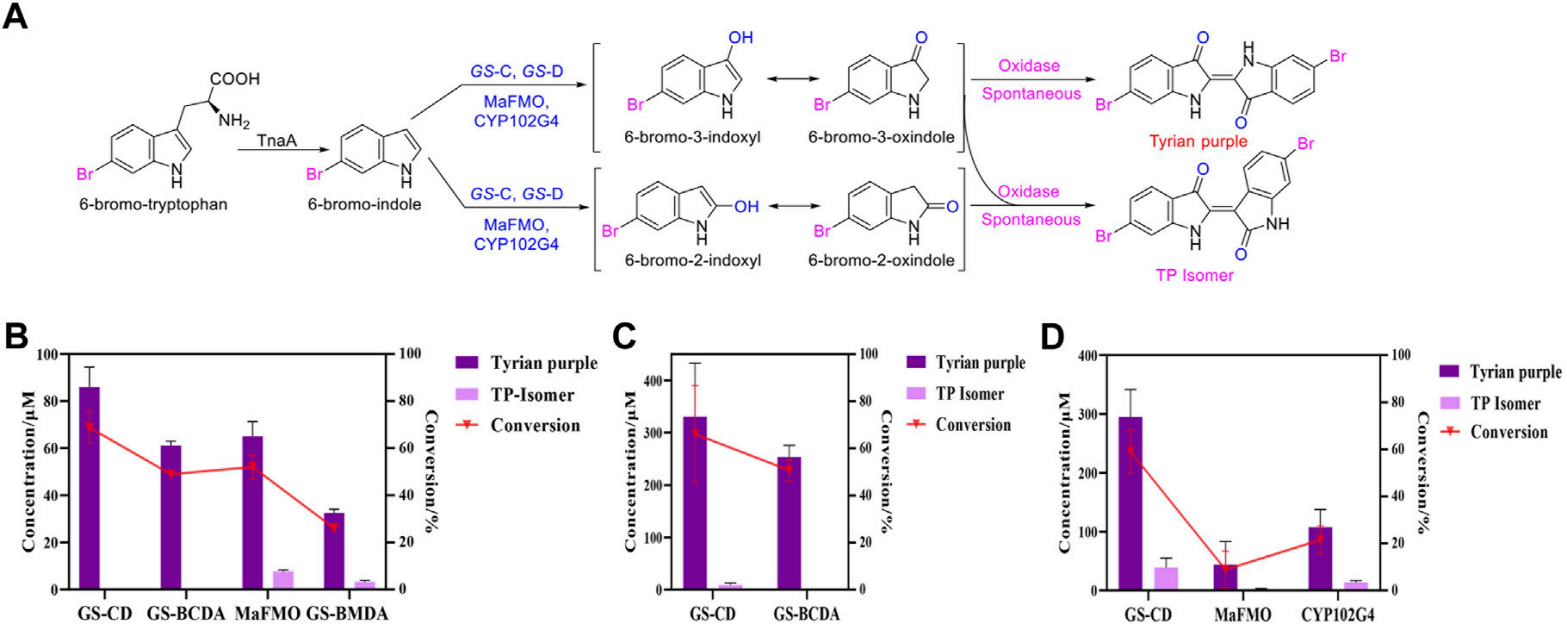
Production of Tyrian purple and TP isomer from 6-Br-tryptophan or 6-Br-indole using *E. coli*, expressing flavin-containing monooxygenase from *Providencia rettgeri* GS-2, *Methylophaga aminisulfidivorans* (MaFMO), or cytochrome P450 enzyme CYP102G4. **(A)** Scheme of synthesis of Tyrian purple and TP isomer from 6-bromo-tryptophan. **(B)** Production and conversion of Tyrian purple by feeding 250 μM 6-Br-indole substrate *in vivo*. **(C)** Production and conversion of Tyrian purple by feeding 1,000 μM 6-Br-indole substrate in whole-cell reaction. **(D)** Production and conversion of Tyrian purple by feeding 1,000 μM 6-Br-tryptophan substrate *in vivo*.

Tyrian purple is an expensive dye and has a promising application in modern times. MaFMO has been reported as an effective oxygenase for Tyrian purple biosynthesis by the oxidation of 6-Br-indole (Lee et al., 2021; Schnepel et al., 2021). The activity of *GS*-C and the indole degradation gene cluster (four *GS* proteins *GS-*A, *GS-*B, *GS-*C, and *GS-*D) contribute to the production of Tyrian purple. We synthesized MaFMO and expressed in *E. coli* BL21 (DE3) with or without the genes *GS*-A, *GS*-B, and *GS*-D in the indole degradation cluster (designed as MaFMO or *GS*-BMDA). We further compared the Tyrian purple and TP isomer production by feeding 250 μM 6-Br-indole substrate in M9 minimal medium. LC-MS-identified *GS-*CD (*GS-*C and *GS-*D) have the highest Tyrian purple production with 85.9 μM and 68.7% conversion ratio *in vivo* (Figure 7B). With *GS-*BA (*GS-*A and *GS-*B), the production of Tyrian purple decreased to 61.2 μM, and the conversion ratio was 49%. Meanwhile, no TP isomer has been detected in the products. The production and conversion ratio of Tyrian purple by MaFMO was 65 μM and 52%, respectively. With *GS-*BA (*GS-*A and *GS-*B), the production of Tyrian purple decreased to 32.5 μM, and the conversion ratio was 26%. Meanwhile, a part of TP isomer has been detected in the products of MaFMO with or without *GS-*BA. These results confirm that the Tyrian purple-producing enzymes *GS*-CD have better activity and selectivity (with *GS-*BA) in the production of Tyrian purple in *E. coli* than MaFMO. When feeding the 1,000 μM 6-Br-indole substrate in a whole-cell reaction (Figure 7C), the *GS-*CD has the highest Tyrian purple production with 330.8 μM and 66.2% conversion ratio in the M9 medium. With *GS-BA*, the production of Tyrian purple decreased by 24% compared with *GS-CD* in the M9 medium. Therefore, by taking advantage of the indole degradation gene cluster in GS-2, we showed that up to 253.4 μM Tyrian purple could be selectively produced from 1,000 μM 6-Br-indole.

As shown in Supplementary Figure 1B, heterologous expression of *GS*-B, *GS*-C, *GS*-D, and *GS*-A from GS-2 in *E. coli* have better production of Tyrian purple than GS-2 when 6-Br-Trp was used as a substrate on a solid plate. To further investigate the biosynthesis of Tyrian purple from 6-Br-Trp in *E. coli*, cytochrome P450 enzyme CYP102G4 (Namgung et al., 2019) was synthesized (CYP102G4 was reported to effectively synthesize indigoid dyes by biotransform indole derivatives). To produce Tyrian purple from 6-Br-Trp, each monooxygenase was co-expressed with TnaA, resulting in three strains, ΔtnaA TnaA + *GS*-CD (designed as *GS*-CD), ΔtnaA TnaA + MaFMO (designed as MaFMO), and ΔtnaA TnaA + CYP102G4 (designed as CYP102G4), and their expression conditions were optimized. When feeding 1,000 μM 6-Br-Trp substrate *in vivo*, compared with MaFMO and CYP102G4, the *GS*-CD has the highest Tyrian purple production with 295.4 μM and 59.1% conversion ratio *in vivo* (Figure 7D). The production and conversion ratio of Tyrian purple by MaFMO were 44.3 μM and 8.9%, respectively. The production and conversion ratio of Tyrian purple by CYP102G4 were 107.5 μM and 21.5%, respectively. Therefore, the flavin-containing monooxygenase *GS*-CD from *Providencia rettgeri* GS-2 is an effective Tyrian purple-producing enzyme, and the indole degradation gene cluster *GS*-BCDA can be used for the selective production of Tyrian purple in *E. coli*.

## 4 Discussion

**FIGURE 8:**
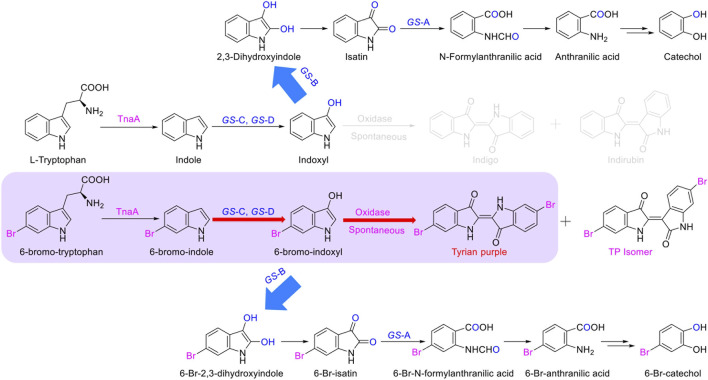
Proposed metabolic pathways contribute to the selectivity Tyrian purple-producing in *E. coli*.

In this study, we identified that the indole biodegradation gene cluster from *Providencia rettgeri* GS-2 could contribute to the selective Tyrian purple production. The molecular mechanisms underpinning this unique performance warrant further investigation. We proposed the indole biodegradation pathway in *Providencia rettgeri* GS-2, which is the catechol pathway according to the degradation products of indole. Notably, the indole biodegradation gene cluster can transform and biodegrade both indole and 6-Br-indole practically. On the one hand, the indole oxygenase *GS*-CD can utilize both indole and 6-Br-indole to form insoluble indigo or Tyrian purple pigment. On the other hand, the oxidations formed by *GS*-CD can be further degraded by *GS*-B and *GS*-A (Figure 8). Based on the *in vivo* and *in vitro* results presented before, *GS*-C and its cofactor-generating enzyme *GS*-D are responsible for the Tyrian purple biosynthesis without observable selectivity toward indole or 6-Br-indole. The indole biodegradation genes *GS*-A and *GS*-B may contribute to the selective Tyrian purple production. Even though we have purified *GS*-A and *GS*-B, it is difficult to obtain the standards of 2,3-dihydroxyindole and N-formylanthranilic acid, the further investigation of the molecular mechanisms of the selective Tyrian purple production would jog along.

**FIGURE 9:**
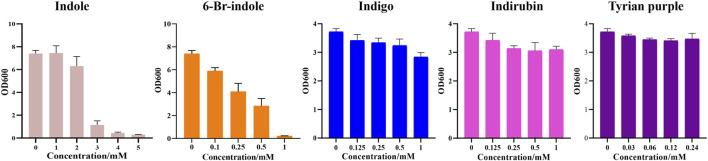
Effect of indole, 6-Br-indole, indigo, indirubin, and Tyrian purple on the growth of *E. coli.*

Furthermore, during our substrate supplement experiments, we found that 6-Br-indole has a significant influence on the growth of *E. coli*. By comparing the effect of indole, 6-Br-indole, indigo, indirubin, and Tyrian purple on the growth of *E. coli*, we found 6-Br-indole has a significant influence on the growth of *E. coli* even at the concentration of 100 μM (Figure 9). The toxicity of indole is above 2 mM. Indigo, indirubin, and Tyrian purple have little effect on the growth of *E. coli*. Thus, the toxicity of 6-Br-indole may drive the faster transformation of 6-Br-indole to non-toxic Tyrian purple. Meanwhile, the catalytic efficiency of *GS*-CD (k_cat_) on 6-Br-indole is faster than that of indole (about two-folds) (Table 5). The transformation of 6-Br-indole to Tyrian purple probably is dominant, and the biodegradation of 6-Br-indole is minor. As the toxicity of indole is much smaller than 6-Br-indole, and the catalytic efficiency of *GS*-CD (k_cat_) on indole is slow, the strain may maintain a balance between indole transformation and degradation, that is, indole was catalyzed by the *GS*-CD to indoxyl, which was rapidly degraded by *GS*-B and *GS*-A, so indigo/indirubin cannot be synthesized. Therefore, we identified Tyrian purple, the common indole transformation product, and indigo and indirubin were not detected when the indole biodegradation gene cluster reaction *in vivo* or *in vitro* took place (Figures 4C, 5).

**TABLE 5:** Steady-state kinetic parameters for NADH oxidation activity on various substrates by *GS*-CD and MaFMO.

Protein	Substrate	K_m_/μm	K_cat_/min	K_cat_/K_m_
*GS*-CD	Indole	217.4	0.05796	0.00027
	6-Br-indole	405.8	0.1264	0.00031
MaFMO	Indole	242.2	0.01475	0.00006
	6-Br-indole	303.65	0.03129	0.00010

**FIGURE 10:**
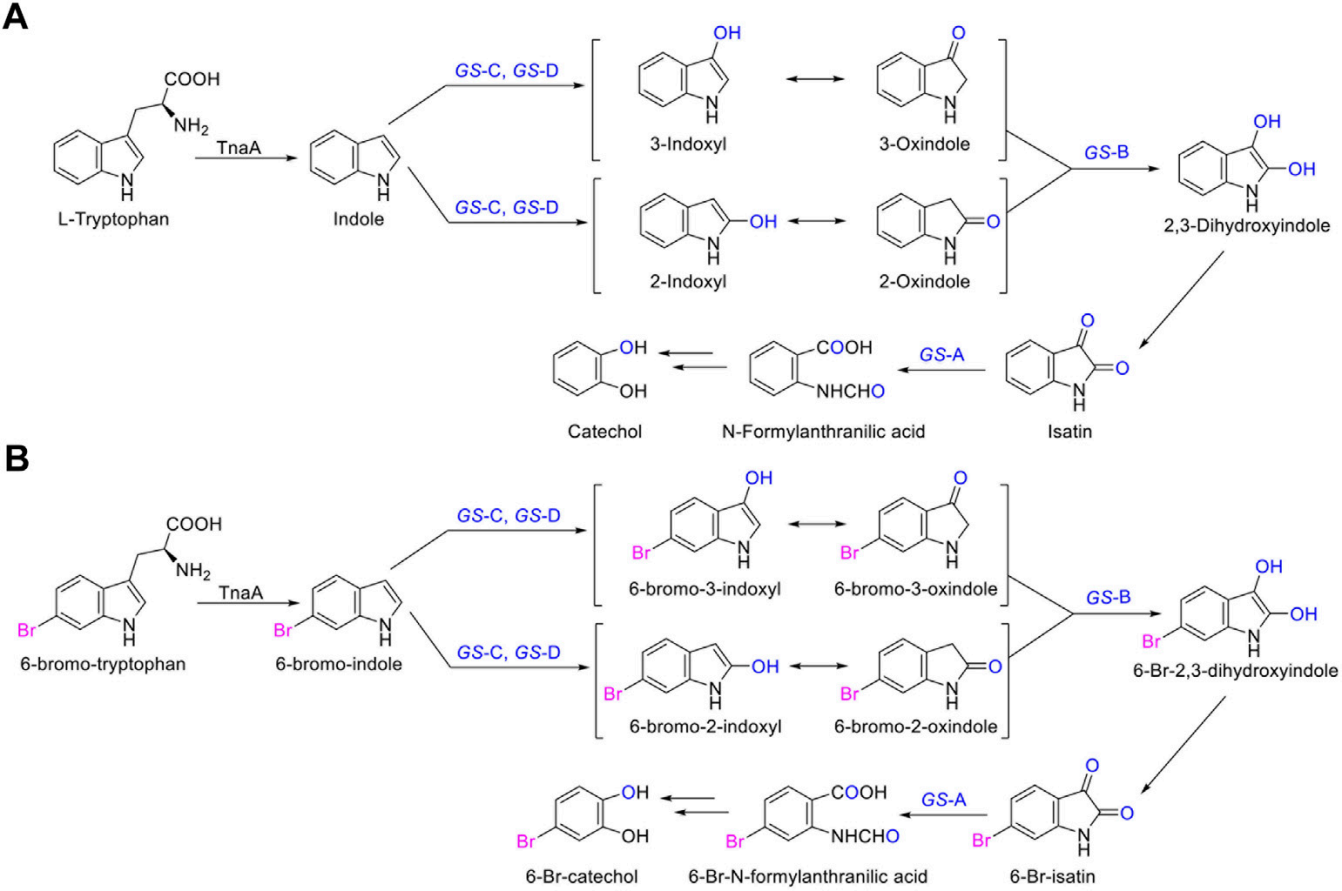
**(A,B)** Scheme of the indole biodegradation gene clusters assisted selective synthesis of Tyrian purple.

In the biosynthesis of Tyrian purple, the formation of TP isomer, which is a stereoisomer of Tyrian purple, is a major side reaction. In addition, the indole oxygenase *GS*-C oxidized the C-2 and C-3 positions of 6-Br-indole, forming the C-2 and C-3 6-Br-indoxyl, which can be an auto-oxidation to Tyrian purple and TP isomer simultaneously (Figure 7). As C2-specific hydroxylation of indole/6-Br-indole leads to the formation of isatin/6-Br-isatin, which further reacts with indoxyl/6-Br-indoxyl and generates the asymmetrical form of indirubin/TP isomer (Cooksey, 2001). In the proposed indole biodegradation pathway, indole biodegradation genes *GS-*B and *GS-*A can degrade C-2 and C-3 oxidations of 6-Br-indole to 6-Br-isatin simultaneously, and further be degraded (Figure 10), but the amount of C-3 oxidation is far more than C-2’s (Figures 4C, 7C). So the C-2 oxidation-related transformation product TP isomer cannot be detected. Thus, the indole biodegradation gene cluster biodegrades isatin/6-Br-isatin contribute to the selective Tyrian purple production.

We also confirmed that the Tyrian purple-producing enzymes *GS*-CD have better activity in the production of Tyrian purple in *E. coli* than the most reported monooxygenase MaFMO. The steady-state kinetic parameters for NADH oxidation activity on various substrates by *GS*-CD and MaFMO proteins were determined (Table 5). The catalytic efficiency of *GS*-CD (k_cat_/K_m_) on indole/6-Br-indole was higher than that of MaFMO. Those results further prove that *GS*-CD have better activity in the production of Tyrian purple in *E. coli* than MaFMO. The effective enzymes *GS*-CD that are found would lay a solid foundation for the promising biosynthesis of Tyrian purple. Incorporating the indole biodegradation gene cluster for the production of Tyrian purple would not only allow obtaining a purer product but also simplify the downstream steps of biosynthesis.

## Data Availability

The datasets presented in this study can be found in online repositories. The names of the repository/repositories and accession number(s) can be found below: NCBI SUB12350614.

## Supplementary material

Supplementary Figure S1 Growth curve and the yields of Tyrian purple-producing strains. **(A)** Growth curve of Tyrian purple-producing strains. **(B)** Yields of Tyrian purple produced by Tyrian purple-producing strains when feeding different substrates on a solid plate. **GS-2**, in the bacterial strain GS-2. ***E. coli***, heterologous expression of *GS*-B, *GS*-C, *GS*-D, and *GS*-A from GS-2 in *E. coli*.

Supplementary Figure S2 Flavin reductase activity of the purified *GS*-D protein.

## References

[B1] Alemayehu D. Gordon L.M. O'mahony M.M. O'leary N.D. Dobson A.D. (2004). Cloning and functional analysis by gene disruption of a novel gene involved in indigo production and fluoranthene metabolism in Pseudomonas alcaligenes PA-10. FEMS Microbiol Lett 239, 285–293. 10.1016/j.femsle.2004.08.046 15476978

[B2] Ameria S.P. Jung H.S. Kim H.S. Han S.S. Kim H.S. Lee J.H. (2015). Characterization of a flavin-containing monooxygenase from Corynebacterium glutamicum and its application to production of indigo and indirubin. Biotechnol Lett 37, 1637–1644. 10.1007/s10529-015-1824-2 25851950

[B3] Doukyu N Aono R. (1997). Biodegradation of indole at high concentration by persolvent fermentation with Pseudomonas sp. ST-200. Extremophiles 1, 100–105. 10.1007/s007920050021 9680309

[B4] Arora P.K. Sharma A. Bae H. (2015). Microbial Degradation of Indole and Its Derivatives. J. Chem. 2015, 1–13. 10.1155/2015/129159

[B5] Bhushan B. Samanta S. K. Jain R. (2000). Indigo production by naphthalene-degrading bacteria. Lett. Appl. Microbiol. 31, 5–9. 10.1046/j.1472-765x.2000.00754.x 10886605

[B6] O’hara C. M. Brenner F. W. Miller J. M. (2000). Classification, Identification, and Clinical Significance of Proteus, Providencia, and Morganella. Clin. Microbiol. Rev. 13 (4), 534–46. 10.1128/CMR.13.4.534 11023955 PMC88947

[B7] Choi K.-Y. (2020). A review of recent progress in the synthesis of bio-indigoids and their biologically assisted end-use applications. Dyes. Pigm 181, 108570. 10.1016/j.dyepig.2020.108570

[B8] Cooksey C.J. (2001). Tyrian Purple 6, 6’-Dibromoindigo and Related Compounds. Molecules 6, 736–769. 10.3390/60900736

[B9] Dai C. Ma Q. Li Y. Zhou D. Yang B. Qu Y. (2019). Application of an efficient indole oxygenase system from Cupriavidus sp. SHE for indigo production. Bioprocess. Biosyst. Eng. 42, 1963–1971. 10.1007/s00449-019-02189-4 31482396

[B10] Doukyu N. Toyoda K. Aono R. (2003). Indigo production by Escherichia coli carrying the phenol hydroxylase gene from Acinetobacter sp strain ST-550 in a water-organic solvent two-phase system. Appl. Microbiol. Biotechnol. 60, 720–725. 10.1007/s00253-002-1187-1 12664152

[B11] Drewlo S. Bramer C.O. Madkour M. Mayer F. Steinbuchel A. (2001). Cloning and expression of a Ralstonia eutropha HF39 gene mediating indigo formation in Escherichia coli. Appl. Environ. Microbiol. 67, 1964–1969. 10.1128/AEM.67.4.1964-1969.2001 11282658 PMC92822

[B12] Fabara A.N. Fraaije M.W. (2020). An overview of microbial indigo-forming enzymes. Appl. Microbiol. Biotechnol. 104, 925–933. 10.1007/s00253-019-10292-5 31834440 PMC6962290

[B13] Fukuoka K. Tanaka K. Ozeki Y. Kanaly R.A. (2015). Biotransformation of indole by Cupriavidus sp. strain KK10 proceeds through N-heterocyclic- and carbocyclic-aromatic ring cleavage and production of indigoids. Int. Biodeterior. Biodegradation. 97, 13–24. 10.1016/j.ibiod.2014.11.007

[B14] Galac M. Lazzaro B. (2012). Comparative genomics of bacteria in the genus Providencia isolated from wild Drosophila melanogaster. BMC Genomics 13, 612. 10.1186/1471-2164-13-612 23145767 PMC3542290

[B15] Głowacki E.D. Voss G. Leonat L. Irimia-Vladu M. Bauer S. Sariciftci N.S. (2012). Indigo and Tyrian Purple - From Ancient Natural Dyes to Modern Organic Semiconductors. Isr. J. Chem. 52, 540–551. 10.1002/ijch.201100130

[B16] Głowacki E.D. Voss G. Sariciftci N.S. (2013). 25th Anniversary Article: Progress in Chemistry and Applications of Functional Indigos for Organic Electronics. Adv. Mater. 25, 6783–6800. 10.1002/adma.201302652 24151199

[B17] Guo C. Quinn J. Sun B. Li Y. (2015). An indigo-based polymer bearing thermocleavable side chains for n-type organic thin film transistors. J. Mater. Chem. C. 3, 5226–5232. 10.1039/c5tc00512d

[B18] Heine T. van Berkel W. J. H. Gassner G. van Pée K. H. Tischler D. (2018). Two-Component FAD-Dependent Monooxygenases: Current Knowledge and Biotechnological Opportunities. Biology 7, 42. 10.3390/biology7030042 30072664 PMC6165268

[B19] Heine T. Grossmann C. Hofmann S. Tischler D. (2019). Indigoid dyes by group E monooxygenases: mechanism and biocatalysis. Biol. Chem. 400, 939–950. 10.1515/hsz-2019-0109 30844759

[B20] Kim J. Lee K. Kim Y. Kim C. Lee K. (2003). Production of dyestuffs from indole derivatives by naphthalene dioxygenase and toluene dioxygenase. Lett Appl Microbiol 36, 343–348. 10.1046/j.1472-765x.2003.01279.x 12753239

[B21] Kim H. Kim G. Song I. Lee J. Abdullah H. Yang C. Oh J.H. (2018). Ambipolar organic phototransistors based on 6, 6'-dibromoindigo. RSC Adv 8, 14747–14752. 10.1039/c8ra02346h 35541344 PMC9079942

[B22] Kim J. Lee J. Lee P.-G. Kim E.-J. Kroutil W. Kim B.-G. (2019). Elucidating Cysteine-Assisted Synthesis of Indirubin by a Flavin-Containing Monooxygenase. ACS Catal 9, 9539–9544. 10.1021/acscatal.9b02613

[B23] Kim M. Lee J.H. Kim E. Choi H. Kim Y. Lee J. (2016). Isolation of Indole Utilizing Bacteria Arthrobacter sp. and Alcaligenes sp. From Livestock Waste. Indian J. Microbiol. 56, 158–166. 10.1007/s12088-016-0570-z 27570307 PMC4984429

[B24] Kwon N.R. Chae J.C. Choi K.Y. Yoo M. Zylstra G.J. Kim Y.M. Kang B.S. Kim E. (2008). Identification of functionally important amino acids in a novel indigo-producing oxygenase from Rhodococcus sp. strain T104. Appl. Microbiol. Biotechnol. 79, 417–422. 10.1007/s00253-008-1445-y 18404265

[B25] Lee J. Kim J. Song J.E. Song W.S. Kim E.J. Kim Y.G. Jeong H.J. Kim H.R. Choi K.Y. Kim B.G. (2021). Production of Tyrian purple indigoid dye from tryptophan in Escherichia coli. Nat. Chem. Biol. 17 (1), 104–112. 10.1038/s41589-020-00684-4 33139950

[B26] Lee J.H. Lee J. (2010). Indole as an intercellular signal in microbial communities. FEMS Microbiol. Rev. 34, 426–444. 10.1111/j.1574-6976.2009.00204.x 20070374

[B27] Li X. Zhang B. Hu Y. Zhao Y. (2021). New Insights Into Gut-Bacteria-Derived Indole and Its Derivatives in Intestinal and Liver Diseases. Front. Pharmacol. 12, 769501. 10.3389/fphar.2021.769501 34966278 PMC8710772

[B28] Lin G.H. Chen H.P. Huang J.H. Liu T.T. Lin T.K. Wang S.J. Tseng C.H. Shu H.Y. (2012). Identification and characterization of an indigo-producing oxygenase involved in indole 3-acetic acid utilization by Acinetobacter baumannii. Antonie. Van. Leeuwenhoek 101 (4), 881–890. 10.1007/s10482-012-9704-4 22311185

[B29] Lin G.H. Chen H.P. Shu H.Y. (2015). Detoxification of Indole by an Indole-Induced Flavoprotein Oxygenase from Acinetobacter baumannii. PLoS One 10 (9), e0138798. 10.1371/journal.pone.0138798 26390211 PMC4577076

[B30] Lu Y. Mei L. (2007). Co-expression of P450 BM3 and glucose dehydrogenase by recombinant Escherichia coli and its application in an NADPH-dependent indigo production system. J. Ind. Microbiol. Biotechnol. 34, 247–253. 10.1007/s10295-006-0193-1 17171348

[B31] Ma Q. Liu Z. Yang B. Dai C. Qu Y. (2019a). Characterization and functional gene analysis of a newly isolated indole-degrading bacterium Burkholderia sp. IDO3. J. Hazard. Mater. 367367, 144144–151151. 10.1016/j.jhazmat.2018.12.068 30594713

[B32] Ma Q. Yang B. Qu H. Gao Z. Qu Y. Sun Y. (2019b). Identification and functional study of an iif2 gene cluster for indole degradation in Burkholderia sp. IDO3. Int. Biodeterior. Biodegradation. 142, 36–42. 10.1016/j.ibiod.2019.04.011

[B33] Ma Q. Zhang X. Qu Y. (2018). Biodegradation and Biotransformation of Indole: Advances and Perspectives. Front. Microbiol. 9, 2625. 10.3389/fmicb.2018.02625 30443243 PMC6221969

[B34] Mcgovern P. Michel R. (1990). Royal purple dye: the chemical reconstruction of the ancient Mediterranean industry. Acc. Chem. Res. 23, 152–158. 10.1021/ar00173a006

[B35] Mendoza-Avila J. Chauhan K. Vazquez-Duhalt R. (2020). Enzymatic synthesis of indigo-derivative industrial Dyes. Pigm 178, 108384. 10.1016/j.dyepig.2020.108384

[B36] Namgung S. Park H.A. Kim J. Lee P.-G. Kim B.-G. Yang Y.-H. Choi K.-Y. (2019). Ecofriendly one-pot biosynthesis of indigo derivative dyes using CYP102G4 and PrnA halogenase. Dyes Pigm 162, 80–88. 10.1016/j.dyepig.2018.10.009

[B37] Ngangbam A.K. Waters D.L.E. Whalan S. Baten A. Benkendorff K. (2015). Indole-Producing Bacteria from the Biosynthetic Organs of a Muricid Mollusc Could Contribute to Tyrian Purple Production. J. Shellfish Res. 34 (2), 443–454. 10.2983/035.034.0228

[B38] O'Connor K. E. Dobson A. D. Hartmans S. (1997). Indigo Formation by Microorganisms Expressing Styrene Monooxygenase Activity. Appl. Environ. Microbiol. 63 (11), 4287–4291. 10.1128/aem.63.11.4287-4291.1997 9361415 PMC168748

[B39] Qu Y. Ma Q. Liu Z. Wang W. Tang H. Zhou J. Xu P. (2017). Unveiling the biotransformation mechanism of indole in a Cupriavidus sp. strain. Mol. Microbiol. 106 (6), 905–918. 10.1111/mmi.13852 28963777

[B40] Qu Y. Shen E. Ma Q. Zhang Z. Liu Z. Shen W. Wang J. Li D. Li H. Zhou J. (2015a). Biodegradation of indole by a newly isolated Cupriavidus sp. SHE. J. Environ. Sci (China). 34, 126–132. 10.1016/j.jes.2015.01.023 26257355

[B41] Qu Y. Shi S. Zhou H. Ma Q. Li X. Zhang X. Zhou J. (2012). Characterization of a Novel Phenol Hydroxylase in Indoles Biotranformation from a Strain Arthrobacter sp. W1. PLoS One 7, e44313. 10.1371/journal.pone.0044313 23028517 PMC3441600

[B42] Qu Y. Zhang Z. Ma Q. Shen E. Shen W. Wang J. Cong L. Li D. Liu Z. Li H. Zhou J. (2015b). Biotransformation of Indole and Its Derivatives by a Newly Isolated Enterobacter sp. M9Z. Appl. Biochem. Biotechnol. 175, 3468–3478. 10.1007/s12010-015-1518-1 25725798

[B43] Sadauskas M Vaitekūnas J Gasparavicˇiūte˙ R Meškys R (2017). Indole Biodegradation in Acinetobacter sp. Strain O153: Genetic and Biochemical Characterization. Appl. Environ. Microbiol. 83 (19), e01453–17. 10.1128/AEM.01453-17 28778892 PMC5601350

[B44] Schnepel C. Dodero V.I. Sewald N. (2021). Novel Arylindigoids by Late-Stage Derivatization of Biocatalytically Synthesized Dibromoindigo. Chem. Eur. J. 27, 5404–5411. 10.1002/chem.202005191 33496351 PMC8048522

[B45] Tomberlin J.K. Crippen T.L. Wu G. Griffin A.S. Wood T.K. Kilner R.M. (2017). Indole: An evolutionarily conserved influencer of behavior across kingdoms. Bioessays 39 (2), 1600203. 10.1002/bies.201600203 28009057

[B46] Wolk J.L. Frimer A.A. (2010). Preparation of Tyrian purple (6, 6'-dibromoindigo): past and present. Molecules 15, 5473–5508. 10.3390/molecules15085473 20714309 PMC6264235

[B47] Yin B. Gu J.-D. Wan N. (2005). Degradation of indole by enrichment culture and Pseudomonas aeruginosa Gs isolated from mangrove sediment. Int. Biodeterior. Biodegradation. 56, 243–248. 10.1016/j.ibiod.2005.10.001

[B48] Zhang X. Jing J. Zhang L. Song Z. Zhou H. Wu M. Qu Y. Liu L. (2018). Biodegradation characteristics and genomic functional analysis of indole‐degrading bacterial strain *Acinetobacter* sp. JW. J. Chem. Technol. Biotechnol. 94, 1114–1122. 10.1002/jctb.5858

[B49] Zhang X. Qu Y. Ma Q. Zhou H. Li X. Kong C. Zhou J. (2013). Cloning and expression of naphthalene dioxygenase genes from Comamonas sp. MQ for indigoids production. Process Biochem 48, 581–587. 10.1016/j.procbio.2013.02.008

[B50] Zhong B. Robinson N.A. Warner R.D. Barrow C.J. Dunshea F.R. Suleria H.a.R. (2020). LC-ESI-QTOF-MS/MS Characterization of Seaweed Phenolics and Their Antioxidant Potential. Mar. Drugs. 18 (6), 331. 10.3390/md18060331 32599953 PMC7344666

